# Supplementary material for: The impact of surgery and oncological treatment on risk of type 2 diabetes onset in patients with colorectal cancer: nationwide cohort study in Denmark
Source: eLife. 2024 Jun 3;12:RP89354. doi: 10.7554/eLife.89354 (PMC11147501; doi:10.7554/eLife.89354)
Supplement: Supplementary file 1. [file elife-89354-supp1.pdf]

Supplementary table 1. Risk of developing T2D after different types of colorectal cancer surgery with and without chemotherapy – adjusted for cancer stage, Model 1 and Model 2

	Adjusted for UICC		Model 1 + UICC		Model 2+UICC	
	HR (95%CI)	*p-value*	HR (95%CI)	*p-value*	HR (95%CI)	*p-value*
**Surgery and chemotherapy**						
Right-No-Chemo	ref					
Right-Chemo	0.98 (0.83;1.15)	0.788	0.93 (0.78;1.11)	0.441	0.97 (0.81;1.16)	0.731
Left-No-Chemo	1.11 (0.98;1.24)	0.097	0.93 (0.81;1.06)	0.269	0.94 (0.83;1.07)	0.374
Left-Chemo	1.03 (0.89;1.19)	0.730	0.88 (0.74;1.03)	0.120	0.93 (0.79;1.09)	0.373
Rectal-No-Chemo	0.93 (0.82;1.05)	0.225	0.81 (0.71;0.93)	0.002	0.85 (0.74;0.97)	0.018
Rectal-Chemo	0.89 (0.76;1.04)	0.139	0.76 (0.64;0.90)	0.002	0.82 (0.69;0.97)	0.022

Right: Right-sided colonic resections; Left: Left-sided colonic resections; Rectal: Rectal resections
No-Chemo: No chemotherapy; Chemo: Chemotherapy

All Colon: All right- and left-sided colonic resections with and without chemotherapy
All Rectal: All rectal resections with and without chemotherapy, and with and without radiation therapy

Model 1: adjusted for year of surgery, age at surgery, sex, BMI
Model 2: adjusted like Model 1 and further adjusted for performance status before surgery assessed by American Society of Anesthesiologist physical scale, smoking and alcohol
